# Lignin biosynthesis perturbations affect secondary cell wall composition and saccharification yield in *Arabidopsis thaliana*

**DOI:** 10.1186/1754-6834-6-46

**Published:** 2013-04-26

**Authors:** Rebecca Van Acker, Ruben Vanholme, Véronique Storme, Jennifer C Mortimer, Paul Dupree, Wout Boerjan

**Affiliations:** 1 Department of Plant Systems Biology, VIB, Technologiepark 927, Gent, 9052, Belgium; 2 Department of Plant Biotechnology and Bioinformatics, Ghent University, Gent, 9052, Belgium; 3 Department of Biochemistry, Cambridge University, Cambridge, CB2 1QW, United Kingdom

**Keywords:** *Arabidopsis thaliana*, Lignin, Cellulose, Matrix polysaccharides, Secondary cell wall, Saccharification

## Abstract

**Background:**

Second-generation biofuels are generally produced from the polysaccharides in the lignocellulosic plant biomass, mainly cellulose. However, because cellulose is embedded in a matrix of other polysaccharides and lignin, its hydrolysis into the fermentable glucose is hampered. The senesced inflorescence stems of a set of 20 *Arabidopsis thaliana* mutants in 10 different genes of the lignin biosynthetic pathway were analyzed for cell wall composition and saccharification yield. Saccharification models were built to elucidate which cell wall parameters played a role in cell wall recalcitrance.

**Results:**

Although lignin is a key polymer providing the strength necessary for the plant’s ability to grow upward, a reduction in lignin content down to 64% of the wild-type level in *Arabidopsis* was tolerated without any obvious growth penalty. In contrast to common perception, we found that a reduction in lignin was not compensated for by an increase in cellulose, but rather by an increase in matrix polysaccharides. In most lignin mutants, the saccharification yield was improved by up to 88% cellulose conversion for the *cinnamoyl-coenzyme A reductase1* mutants under pretreatment conditions, whereas the wild-type cellulose conversion only reached 18%. The saccharification models and Pearson correlation matrix revealed that the lignin content was the main factor determining the saccharification yield. However, also lignin composition, matrix polysaccharide content and composition, and, especially, the xylose, galactose, and arabinose contents influenced the saccharification yield. Strikingly, cellulose content did not significantly affect saccharification yield.

**Conclusions:**

Although the lignin content had the main effect on saccharification, also other cell wall factors could be engineered to potentially increase the cell wall processability, such as the galactose content. Our results contribute to a better understanding of the effect of lignin perturbations on plant cell wall composition and its influence on saccharification yield, and provide new potential targets for genetic improvement.

## Background

Since the industrial revolution, mankind has exploited fossil energy sources for manufacturing and transport. Depletion of petroleum reserves, geopolitical tension, and climate change have increased the need for alternative and sustainable sources of energy [1]. One of the potential alternatives, besides solar radiation and wind, is lignocellulosic biomass of which the sugar fraction in the secondary cell wall (cellulose and hemicelluloses) can be used for the production of liquid biofuels, such as bioethanol [2]. However, the enzymatic processing of plant biomass into fermentable sugars, called saccharification, is hampered by the complexity of the secondary cell wall structure and the presence of lignin [3].

**Figure 1:**
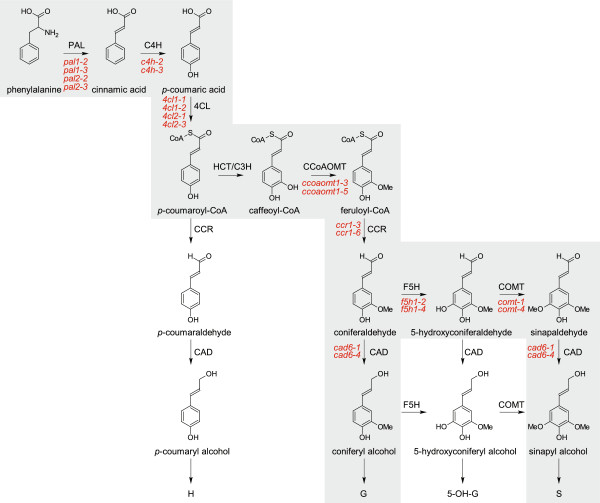
**Phenylpropanoid and monolignol biosynthetic pathways.** The grey box represents the phenylpropanoid and monolignol biosynthetic pathways that are generally accepted for angiosperms with indication of the 20 mutant alleles studied (in red). The general phenylpropanoid pathway starts with *PAL* and ends with *CCoAOMT*, whereas the monolignol-specific biosynthesis starts with *CCR*.

The major component of the secondary cell wall is cellulose, a polymer of 1,4-linked β-d-glucose units, of which the largest fraction is organized into microfibrils through inter- and intramolecular hydrogen bonds and van der Waals forces. The fraction of the microfibril-bound cellulose is called crystalline cellulose to distinguish it from the remaining ‘unorganized’ cellulose, called amorphous cellulose [4]. The spaces between individual cellulose microfibrils are largely filled with hemicelluloses that are far more complex in sugar composition and linkage types than cellulose. In angiosperms, the major hemicelluloses are glucuronoxylans, xyloglucans, and glucomannans [5]. Glucuronoxylans consist of a linear backbone of 1,4-linked β-d-xylopyranosyl units that are substituted with acetyl and 4-*O*-methylglucuronic acid side chains. Xyloglucans are made of a d-glucose backbone of which 75% of the residues are substituted by d-xylose. In addition, l-arabinose and d-galactose residues can be attached to the xylose residues [6]. Glucomannans occur in minor amounts in the secondary cell wall of hardwoods and are linear chains of glucose and mannose residues [5]. The third major type of polymer in the secondary cell wall is lignin. In dicotyledonous plants, lignin is mainly made from the monolignols coniferyl alcohol and sinapyl alcohol and traces of *p*-coumaryl alcohol that give rise to guaiacyl (G), syringyl (S), and *p*-hydroxyphenyl (H) units. Most of these units are linked via ether bonds (in so-called β–O–4-structures) and carbon-carbon bonds [in resinol (β–β), and phenylcoumaran (β–5) structures] [7,8]. The monolignols are synthesized from phenylalanine through the general phenylpropanoid and monolignol-specific pathways (Figure 1). After the monolignols are transported to the cell wall, they are oxidized by laccases and peroxidases to monolignol radicals that then couple in a combinatorial fashion, finally generating the lignin polymer.

To tailor biomass for improved cell wall deconstruction, a profound knowledge is required of the factors that determine the recalcitrance of cell walls to saccharification [9]. One of the major factors causing recalcitrance to saccharification is the presence of lignin, as shown in a series of transgenic alfalfa (*Medicago sativa*) lines with variable lignin amount and composition [3] and, later on, in tobacco (*Nicotiana tabacum*) [10], maize (*Zea mays*) [11], switchgrass (*Panicum virgatum*) [12-14], sugarcane (*Saccharum* sp.) [15], sorghum (*Sorghum bicolor*) [16], brachypodium (*Brachypodium distachyon*) [17], poplar (*Populus* sp.) [18-21], and eucalyptus (*Eucalyptus globulus*) [22]. Besides the lignin amount, the lignin composition affects saccharification as well [19,22-24]. A few studies also pointed to the influence of cell wall polysaccharides: cellulose crystallinity [22,25,26], hemicellulose amount and composition [10,26,27], and perhaps xylan branching [28]. Cell wall engineering is complicated by our limited knowledge about potential crosstalk between the biosynthetic pathways of the main cell wall polymers. For example, several reports have suggested that a reduced lignin amount is compensated for by an increase in cellulose, as observed in poplars down-regulated in 4-coumarate:CoA ligase (*4CL*) and caffeic acid *O*-methyltransferase (*COMT*) [29-31]. However, in an in-depth study of a series of lignin-deficient *Arabidopsis thaliana* mutants, no such correlation was found in any of these mutants [32].

To investigate whether lignin deficiency alters the level or composition of cell wall polysaccharides, we analyzed the amount and composition of the three main cell wall polymers (lignin, cellulose, and matrix polysaccharides) of the senesced inflorescence stems of *Arabidopsis* mutants, mutated in 10 different genes of the phenylpropanoid and monolignol biosynthetic pathways [32], i.e., two mutant alleles of each gene encoding phenylalanine ammonia lyase 1 (*PAL1*), *PAL2*, cinnamate 4-hydroxylase (*C4H*), *4CL1*, *4CL2*, caffeoyl-CoA *O*-methyltransferase 1 (*CCOAOMT1*), cinnamoyl-CoA reductase 1 (*CCR1*), ferulate 5-hydroxylase 1 (*F5H1*), *COMT*, and cinnamyl alcohol dehydrogenase 6 (*CAD6*). In addition, we developed a small-scale saccharification protocol for *Arabidopsis* inflorescence stems and analyzed whether the altered cell wall composition in the mutants affected the saccharification yield. Analysis by whole cell wall Nuclear Magnetic Resonance (NMR) methods provided comparable findings on the lignin composition (see Chylla *et al*., accompanying paper). The high number of mutants and biological replicates allowed us to draw correlations among cell wall composition and saccharification yield and to design a statistical model for the saccharification yield as a function of the cell wall composition.

## Results

### Biomass of *Arabidopsis* lignin mutants

For 10 genes of the phenylpropanoid and monolignol biosynthetic pathways, two mutant alleles (*pal1-2*, *pal1-3*, *pal2-2*, *pal2-3*, *c4h-2*, *c4h-3*, *4cl1-1*, *4cl1-2*, *4cl2-1*, *4cl2-3*, *ccoaomt1-3*, *ccoaomt1-5*, *ccr1-3*, *ccr1-6*, *f5h1-2*, *f5h1-4*, *comt-1*, *comt-4*, *cad6-1*, and *cad6-4*[32]) were grown in 16 biological replicates in a controlled growth room, together with 32 replicates of the wild-type. To compare the cell wall composition and saccharification yield, these mutants should ideally develop similarly as the wild-type. Because perturbations in the lignin biosynthesis often affected plant growth, we first compared the final height and weight of the senesced inflorescence stems of the mutants with those of the wild-type (Table 1). Most mutants were comparable to wild-type, with a few exceptions. As anticipated, developmental defects were noticed for *c4h-2*, *ccr1-3*, and *ccr1-6*, of which the final height of their inflorescence stems was reduced by 29%, 83%, and 34% as compared to the wild-type, respectively [32-34]. Notably, the *ccr1-3* mutant had a bushy appearance with a small primary inflorescence (that died early) and many secondary inflorescences. The strongest reduction in biomass, as measured by the weight of the primary senesced inflorescence stem (without rosette leaves, axillary inflorescences, and siliques), was seen for *c4h-2*, *ccr1-3*, and *ccr1-6*, as expected, but also for *ccoaomt1-3*. The weight of their inflorescence stem was reduced by 52%, 77%, 29%, and 31% compared to the wild-type, respectively.

**Table 1:** Phenotypic traits

**Line**	**Height (cm)**	**Mass (mg)**	**% CWR**
WT Col-0	49.6 ± 3.7	64.3 ± 9.9	78.4 ± 1.7
*pal1-2*	49.4 ± 3.0	63.4 ± 13.5	76.6 ± 1.9
*pal1-3*	49.3 ± 3.5	57.5 ± 10.3	76.3 ± 3.5
*pal2-2*	48.8 ± 2.4	62.5 ± 9.5	77.7 ± 1.8
*pal2-3*	45.6 ± 2.9	54.6 ± 14.4	76.7 ± 1.8
*c4h-2*	35.4 ± 4.9*	31.1 ± 8.6*	76.6 ± 2.3
*c4h-3*	49.9 ± 2.8	55.9 ± 12.9	78.7 ± 1.8
*4cl1-1*	49.2 ± 2.0	63.4 ± 8.5	79.1 ± 2.4
*4cl1-2*	49.2 ± 2.7	62.5 ± 8.2	78.3 ± 2.3
*4cl2-1*	47.9 ± 3.8	59.6 ± 13.5	77.3 ± 0.8
*4cl2-3*	48.2 ± 3.9	62.9 ± 15.8	77.2 ± 3.0
*ccoaomt1-3*	45.9 ± 4.9	44.5 ± 10.2*	77.8 ± 3.3
*ccoaomt1-5*	45.9 ± 2.4	57.6 ± 11.7	77.1 ± 2.5
*ccr1-3*	8.6 ± 1.9*	14.9 ± 1.5*	67.1 ± 4.0*
*ccr1-6*	32.5 ± 2.3*	45.4 ± 6.5*	72.6 ± 2.9*
*f5h1-2*	45.0 ± 1.8	59.3 ± 8.6	78.7 ± 1.6
*f5h1-4*	47.1 ± 4.2	60.5 ± 18.3	78.6 ± 2.1
*comt-1*	47.4 ± 3.1	56.3 ± 13.4	76.7 ± 1.7
*comt-4*	47.4 ± 6.7	58.4 ± 13.3	77.0 ± 2.3
*cad6-1*	51.2 ± 2.2	63.1 ± 9.6	80.0 ± 2.3
*cad6-4*	46.1 ± 2.6	54.5 ± 10.1	78.2 ± 1.7

Height and biomass were determined on fully senesced primary inflorescence stems (free of rosette leaves, axillary inflorescences, and siliques). The data represent the average value of 16 biological repeats for the mutants and 32 biological repeats for the wild-type (WT) (± SD). The cell wall residue (CWR) (expressed as % dry weight) was determined gravimetrically after a sequential extraction (mutants, n = 8; wild-type, n = 16). Decreases compared to the wild-type are in italics. * *p* < 0.001 (Dunnett-Hsu adjusted *t*-test).

Prior to determining the cell wall composition, the senesced inflorescence stems were pooled two by two and a crude cell wall residue (CWR) of the dry stems was prepared through a sequential extraction with water, ethanol, chloroform, and acetone. Based on the weight loss by this sequential extraction, the dry stem biomass of the wild-type was calculated to be composed of approximately 78% CWR (Table 1). Similar values were obtained for the different mutants, except for *ccr1-3* and *ccr1-6*, which had 14% and 7% less CWR (and thus more extractables) than the wild-type. For convenience, all further data are expressed on a CWR basis.

### Lignin amount and composition

The lignin content was measured spectrophotometrically by the acetyl bromide (AcBr) method, adapted for small samples sizes (modified from [35]). Compared to the wild-type, the lignin content of both mutant alleles in *C4H*, *4CL1*, *CCoAOMT1*, and *CCR1* had decreased severely (Table 2). The strongest reduction was found for *c4h-2* (−59%), *c4h-3* (−36%), *ccr1-3* (−51%), and *ccr1-6* (−52%), whereas it was more moderate for the *4cl1* and *ccoaomt1* mutants (between 21 and 26%).

**Table 2:** Lignin content and composition

**Line**	**AcBr**	**H + G + S**	**% H**	**% G**	**% S**	**% 5-OH-G**	**%*bis*-β–O–4-FA**	**% β–O–4-FA-I**	**% β–O–4-FA-II**	**% G aldehydes**	**% S aldehydes**	**S/G**
WT Col-0	14.5 ± 0.6	817.7 ± 204.7	0.5 ± 0.1	70.3 ± 1.3	29.0 ± 1.4	0.11 ± 0.03	0.07 ± 0.03	0.02 ± 0.008	0.01 ± 0.005	0.02 ± 0.036	0.01 ± 0.006	0.41 ± 0.03
*pal1-2*	14.0 ± 0.6	793.4 ± 186.8	0.5 ± 0.1	67.0 ± 2.3	32.3 ± 2.3	0.12 ± 0.03	0.06 ± 0.03	0.02 ± 0.004	0.01 ± 0.005	0.01 ± 0.028	0.02 ± 0.009	0.48 ± 0.05
*pal1-3*	13.9 ± 0.8	812.4 ± 216.4	0.5 ± 0.2	67.7 ± 2.0	31.5 ± 2.0	0.11 ± 0.03	0.05 ± 0.03	0.02 ± 0.007	0.01 ± 0.002	0.02 ± 0.032	0.01 ± 0.005	0.47 ± 0.04
*pal2-2*	14.1 ± 1.0	821.7 ± 164.3	0.5 ± 0.2	70.3 ± 2.3	29.0 ± 2.2	0.10 ± 0.02	0.07 ± 0.02	0.02 ± 0.006	0.01 ± 0.004	0.02 ± 0.034	0.02 ± 0.008	0.41 ± 0.04
*pal2-3*	14.2 ± 0.9	818.8 ± 233.5	0.4 ± 0.1	69.2 ± 2.1	30.2 ± 2.0	0.10 ± 0.04	0.05 ± 0.02	0.02 ± 0.009	0.01 ± 0.005	0.03 ± 0.035	0.01 ± 0.011	0.44 ± 0.04
*c4h-2*	6.0 ± 0.4**	530.7 ± 160.8**	1.9 ± 0.8**	55.7 ± 2.6**	42.1 ± 2.5**	0.15 ± 0.03	0.10 ± 0.07	0.03 ± 0.012	0.02 ± 0.002	0.02 ± 0.020	0.04 ± 0.027	0.76 ± 0.08**
*c4h-3*	9.3 ± 0.3**	870.7 ± 248.7	0.7 ± 0.2	53.5 ± 2.1**	45.6 ± 2.1**	0.13 ± 0.02	0.08 ± 0.03	0.02 ± 0.008	0.01 ± 0.002	0.01 ± 0.026	0.03 ± 0.026	0.86 ± 0.07**
*4cl1-1*	10.8 ± 0.5**	828.5 ± 197.4	0.8 ± 0.3	59.9 ± 1.9**	39.1 ± 2.0**	0.12 ± 0.02	0.10 ± 0.04	0.02 ± 0.008	0.01 ± 0.003	0.00 ± 0.002	0.03 ± 0.023	0.65 ± 0.05**
*4cl1-2*	10.8 ± 0.7**	804.4 ± 151.9	0.8 ± 0.2	60.6 ± 2.7**	38.3 ± 2.1**	0.11 ± 0.05	0.10 ± 0.04	0.02 ± 0.010	0.01 ± 0.004	0.02 ± 0.055	0.02 ± 0.014	0.63 ± 0.06**
*4cl2-1*	14.3 ± 0.7	783.3 ± 169.1	0.5 ± 0.2	69.6 ± 2.3	29.6 ± 2.3	0.12 ± 0.02	0.07 ± 0.03	0.02 ± 0.007	0.01 ± 0.003	0.02 ± 0.040	0.01 ± 0.004	0.43 ± 0.05
*4cl2-3*	14.0 ± 0.5	819.7 ± 179.8	0.5 ± 0.1	70.9 ± 1.7	28.3 ±1.8	0.12 ± 0.03	0.08 ± 0.03	0.02 ± 0.006	0.01 ± 0.004	0.00 ± 0.004	0.01 ± 0.009	0.40 ± 0.04
*ccoaomt1-3*	11.4 ± 1.7**	789.3 ± 156.8	0.6 ± 0.1	62.3 ± 4.4**	36.7 ± 4.3**	0.13 ± 0.03	0.14 ± 0.06	0.04 ± 0.010	0.02 ± 0.008	0.02 ± 0.029	0.01 ± 0.011	0.60 ± 0.11**
*ccoaomt1-5*	10.9 ± 0.4**	696.1 ± 187.9	0.9 ± 0.3	60.7 ± 2.9**	38.0 ± 3.0**	0.12 ± 0.03	0.14 ± 0.03	0.08 ± 0.113	0.03 ± 0.023	0.03 ± 0.036	0.02 ± 0.007	0.63 ± 0.08**
*ccr1-3*	7.1 ± 0.7**	192.1 ± 49.3**	4.5 ± 1.4**	71.7 ± 4.9	18.5 ± 5.0**	0.23 ± 0.11	2.83 ± 1.91**	1.38 ± 0.569**	0.81 ± 0.218**	0.04 ± 0.030	0.09 ± 0.048**	0.26 ± 0.09**
*ccr1-6*	6.9 ± 0.4**	229.3 ± 79.0**	1.2 ± 1.2*	68.7 ± 2.6	24.9 ± 2.7**	0.17 ± 0.08	2.55 ± 1.04**	1.61 ± 0.366**	0.94 ± 0.317**	0.05 ± 0.061	0.06 ± 0.042	0.36 ± 0.05
*f5h1-2*	15.9 ± 0.6*	596.0 ± 167.4**	0.4 ± 0.1	99.3 ± 0.2**	0.0 ± 0.0**	0.03 ± 0.01	0.10 ± 0.05	0.04 ± 0.013	0.02 ± 0.008	0.02 ± 0.025	0.01 ± 0.009	0.00 ± 0.00**
*f5h1-4*	15.3 ± 0.1	595.6 ± 137.0**	0.4 ± 0.1	99.3 ± 0.2**	0.0 ± 0.0**	0.03 ± 0.01	0.08 ± 0.04	0.04 ± 0.012	0.03 ± 0.007	0.02 ± 0.031	0.01 ± 0.003	0.00 ± 0.00**
*comt-1*	13.7 ± 1.0	499.6 ± 151.0**	0.7 ± 0.2	97.7 ± 0.6**	0.5 ± 0.1**	0.93 ± 0.32**	0.09 ± 0.04	0.03 ± 0.012	0.02 ± 0.009	0.05 ± 0.070	0.02 ± 0.025	0.01 ± 0.00**
*comt-4*	14.1 ± 0.9	502.8 ± 177.2**	0.7 ± 0.3	94.9 ± 0.6**	3.2 ± 0.4**	0.94 ± 0.35**	0.10 ± 0.02	0.04 ± 0.016	0.02 ± 0.018	0.05 ± 0.079	0.01 ± 0.007	0.03 ± 0.00**
*cad6-1*	14.5 ± 0.8	779.7 ± 183.7	0.3 ± 0.1	73.7 ± 2.1*	25.5 ± 2.1*	0.10 ± 0.02	0.10 ± 0.04	0.02 ± 0.006	0.01 ± 0.004	0.04 ± 0.061	0.16 ± 0.132**	0.35 ± 0.04
*cad6-4*	14.4 ± 0.7	742.0 ± 206.3	0.3 ± 0.1	75.3 ± 4.2**	23.9 ± 4.3**	0.16 ± 0.14	0.09 ± 0.02	0.03 ± 0.008	0.01 ± 0.005	0.02 ± 0.030	0.18 ± 0.122**	0.32 ± 0.07**

Lignin content and composition for the 20 lignin mutants (n = 8) and wild-type (WT) (n = 16) (± SD). Lignin content was determined with the AcBr assay and expressed as percentage CWR. Lignin composition was determined with thioacidolysis. The sum of H, G, and S is expressed in μmol g^-1^ AcBr lignin. The relative proportions of the different lignin units were calculated based on the total thioacidolysis yield (including the minor nonconventional lignin units). S/G was calculated based on the absolute values for S and G (expressed in μmol g^-1^ AcBr lignin). *bis*-β–O–4-FA: G-CHR-CHR_2_; β–O–4-FA-I: G-CH = CH-COOH; β–O–4-FA-II: G-CHR-CH_2_-COOH. Increases and decreases compared to the wild-type are in bold and italics, respectively. * 0.001 < *p* < 0.01; ** *p* < 0.001 (Dunnett-Hsu adjusted *t*-test).

Subsequently, the lignin composition was analyzed by thioacidolysis (Table 2) that quantifies the H, G, and S units that are linked by β–O–4-ether bonds in the lignin polymer. The sum of H, G, and S is a good estimate of the total thioacidolysis yield and, thus, the condensation degree of the lignin polymer. The thioacidolysis yields of both mutant alleles of *CCR1*, *ccr1-3* and *ccr1-6*, were reduced by 76% and 72%. Less strong (but nevertheless significant) decreases in thioacidolysis yield were noticed for both *f5h1* and *comt* mutants and for *c4h-2*. The H units were barely detectable in the wild-type and comprised only 0.5% of the total identified thioacidolysis-released units. Only the lignin in *c4h-2* and the two mutant alleles of *CCR1* had a relative increase in thioacidolysis-released H units. The relative amounts of thioacidolysis-released G units (% G units) were reduced in both mutant alleles of *C4H*, *4CL1*, and *CCoAOMT1* and, except for the *ccr1* mutants, increased in other mutants of the monolignol-specific pathway. The relative amount of thioacidolysis-released S units (% S units) followed an inverse relation with the G units: increased in *C4H*, *4CL1*, and *CCoAOMT1* and decreased in all mutants of the monolignol-specific pathway. Consequently, the S/G ratio, typically used to characterize the lignin composition, was increased for both mutant alleles of *C4H*, *4CL1*, and *CCoAOMT1*, whereas it decreased in *ccr1-3*, *cad6-4*, and both mutant alleles of *F5H1* and *COMT*.

In addition to the traditional lignin units (H, G, and S), a number of minor “nonconventional” units were identified and quantified. Although the trace amounts of the 5-hydroxyguaiacyl (5-OH-G) units (derived from the incorporation of 5-hydroxyconiferyl alcohol into lignin) in wild-type plants are actual artefacts of the thioacidolysis procedure, the relative amount of the 5-OH-G units had increased in the *comt* mutants, consistent with previous reports of increased 5-OH-G units in *COMT*-deficient plants [31,36-39]. Units derived from the incorporation of coniferaldehyde and sinapaldehyde (the G and S aldehyde units) could be detected via thioacidolysis markers as previously described [40,41]. Mutants in the last step of the monolignol-specific pathway, *CAD6*, were characterized by a higher incorporation of S aldehydes than of G aldehyde units into the lignin polymer, in line with the higher substrate specificity of CAD6 for sinapaldehyde [42]. Finally, thioacidolysis released three different units derived from the incorporation of ferulic acid (FA) that is also a known minor constituent of lignin [43], two of which were linked via conventional β–O–4-structures (the β–O–4-FA-I and β–O–4-FA-II units) and the third derived from the *bis*-β–O–4-coupling of FA (the *bis*-β–O–4-FA unit) that results in a truncated side chain [43]. Of these three units, the relative abundance of *bis*-β–O–4-FA was the highest. The relative abundance of β–O–4-FA-I, β–O–4-FA-II, and *bis*-β–O–4-FA units had increased in *ccr1* mutants, in agreement with previously reported results of *CCR*-deficient plants [43,44].

### Is lignin modification associated with altered cell wall polysaccharide amount and composition?

To investigate whether perturbations in the lignin biosynthetic pathway also affected the abundance of the other cell wall polymers, we measured the cellulose content with the spectrophotometric phenol-sulfuric acid assay (adapted from [45,46]). In the adapted protocol, the CWR was hydrolyzed with trifluoroacetic acid (TFA) that extracts matrix polysaccharides, but also amorphous cellulose. Therefore, the data presented in Table 3 are estimates of the crystalline cellulose fraction. In *Arabidopsis* mutants deficient in the monolignol-specific pathway, from *CCR1* through *COMT*, the decrease in crystalline cellulose content was the strongest for *ccr1-3* (−40%) and *ccr1-6* (−21%) whereas the mutant alleles of *F5H1* and *COMT* had reductions of between 14% and 19%. All other mutants had similar crystalline cellulose contents to wild-type.

**Table 3:** Polysaccharide content and composition

**Line**	**Cellulose**	**Matrix polysaccharides**	**Rhamnose**	**Fucose**	**Arabinose**	**Xylose**	**Mannose**	**Glucose**	**Galactose**	**Frequency of [Me]GlcA branching of xylan**	**% methylated GlcA on xylan**
WT Col-0	38.9 ± 2.3	35.6 ± 1.4	3.4 ± 0.1	2.2 ± 0.4	12.7 ± 0.4	58.7 ± 1.3	2.8 ± 0.2	5.7 ± 1.7	14.5 ± 0.3	13.3 ± 0.6	64.9 ± 0.6
*pal1-2*	41.6 ± 5.4	35.7 ± 1.8	3.0 ± 0.3**	2.0 ± 0.5	**13.2 ± 0.4**	55.2 ± 3.9*	2.3 ± 0.3*	9.8 ± 4.8	14.5 ± 0.4	13.2 ± 0.9	66.3 ± 0.8
*pal1-3*	42.9 ± 4.0	37.3 ± 1.1	3.3 ± 0.2	2.3 ± 0.3	12.7 ± 0.7	56.0 ± 3.0	2.6 ± 0.2	8.9 ± 4.2	14.2 ± 0.5	13.2 ± 1.0	66.8 ± 1.0
*pal2-2*	41.5 ± 4.6	37.1 ± 1.5	3.5 ± 0.1	2.3 ± 0.3	12.6 ± 0.8	57.3 ± 2.7	2.9 ± 0.2	7.1 ± 4.3	14.4 ± 0.8	13.2 ± 0.6	64.2 ± 1.0
*pal2-3*	41.4 ± 2.5	36.8 ± 1.7	3.4 ± 0.2	2.3 ± 0.2	12.3 ± 0.8	56.7 ± 3.4	2.7 ± 0.3	8.7 ± 5.1	13.9 ± 0.7	13.1 ± 0.9	64.6 ± 1.1
*c4h-2*	36.3 ± 2.1	42.9 ± 1.7**	3.6 ± 0.1	2.7 ± 0.4*	15.4 ± 0.9**	57.0 ± 1.9	2.8 ± 0.1	3.1 ± 0.2	15.4 ± 0.9*	12.6 ± 0.5	80.3 ± 1.2**
*c4h-3*	40.7 ± 3.0	38.7 ± 0.9**	3.3 ± 0.3	2.5 ± 0.4	14.4 ± 0.9**	59.0 ± 1.2	2.8 ± 0.4	2.7 ± 0.6	15.5 ± 1.0*	12.9 ± 0.6	72.1 ± 1.5**
*4cl1-1*	41.4 ± 2.6	37.8 ± 1.4**	3.5 ± 0.2	2.3 ± 0.5	13.3 ± 0.4	58.9 ± 1.5	2.9 ± 0.2	4.3 ± 1.3	14.8 ± 0.3	13.0 ± 0.4	74.7 ± 1.8**
*4cl1-2*	40.7 ± 2.8	39.4 ± 1.2**	3.6 ± 0.1	2.2 ± 0.4	12.4 ± 0.7	58.6 ± 1.6	3.0 ± 0.1	6.2 ± 1.8	14.1 ± 0.6	13.1 ± 1.0	74.1 ± 1.5**
*4cl2-1*	37.8 ± 2.2	38.9 ± 1.4**	3.7 ± 0.3**	2.3 ± 0.2	12.1 ± 1.0	56.7 ± 3.3	2.8 ± 1.1	8.5 ± 6.5	13.8 ± 0.9	12.8 ± 0.4	64.5 ± 0.6
*4cl2-3*	38.5 ± 2.3	38.3 ± 1.3**	3.7 ± 0.2**	2.1 ± 0.4	12.0 ± 0.4	57.4 ± 2.5	3.1 ± 0.2	7.6 ± 2.9	14.0 ± 0.3	12.7 ± 0.6	63.8 ± 1.2
*ccoaomt1-3*	39.8 ± 1.7	39.6 ± 1.4**	3.8 ± 0.2**	2.4 ± 0.1	12.4 ± 0.6	59.6 ± 1.2	3.2 ± 0.2	3.8 ± 0.8	14.9 ± 0.4	13.4 ± 0.7	68.8 ± 2.1**
*ccoaomt1-5*	41.0 ± 2.6	38.4 ± 1.4**	3.4 ± 0.1	2.2 ± 0.4	13.5 ± 1.4	57.1 ± 5.1	3.0 ± 0.6	5.0 ± 2.9	15.8 ± 1.4**	13.5 ± 0.4	73.5 ± 0.6**
*ccr1-3*	23.4 ± 3.5**	46.6 ± 1.5**	3.1 ± 0.3	1.8 ± 0.4	19.2 ± 1.3**	50.3 ± 2.2**	2.4 ± 0.3	4.9 ± 3.4	18.2 ± 1.0**	14.2 ± 0.4	55.2 ± 1.1**
*ccr1-6*	30.9 ± 2.6**	44.6 ± 1.3**	3.6 ± 0.3	2.2 ± 0.5	15.7 ± 0.4**	54.1 ± 1.9**	2.7 ± 0.1	5.6 ± 2.4	16.0 ± 0.5*	14.5 ± 0.4	54.9 ± 1.8**
*f5h1-2*	31.7 ± 2.0**	35.7 ± 1.3	3.6 ± 0.1	2.1 ± 0.5	12.6 ± 0.4	57.0 ± 1.9	3.0 ± 0.1	7.4 ± 2.2	14.4 ± 0.2	13.0 ± 0.7	66.9 ± 0.6
*f5h1-4*	32.8 ± 3.2**	35.8 ± 1.3	3.5 ± 0.2	2.0 ± 0.4	12.5 ± 0.9	55.6 ± 3.7	2.8 ± 0.3	9.2 ± 5.5	14.4 ± 0.7	13.0 ± 0.3	66.3 ± 0.7
*comt-1*	33.4 ± 1.8**	37.2 ± 1.7	3.4 ± 0.2	2.1 ± 0.4	12.7 ± 0.7	56.5 ± 2.6	2.9 ± 0.2	7.6 ± 3.6	14.8 ± 0.4	12.7 ± 0.4	65.6 ± 1.9
*comt-4*	33.2 ± 2.1**	36.1 ± 0.9	3.2 ± 0.1	2.2 ± 0.4	12.8 ± 0.5	55.2 ± 2.6*	2.8 ± 0.2	7.9 ± 3.4	15.9 ± 0.5**	13.1 ± 0.8	65.2 ± 0.6
*cad6-1*	40.6 ± 3.2	34.8 ± 1.2	3.2 ± 0.1	2.3 ± 0.5	13.5 ± 0.9	58.8 ± 1.6	2.6 ± 0.1	5.1 ± 2.7	14.5 ± 0.5	13.0 ± 0.5	66.2 ± 0.7
*cad6-4*	38.9 ± 3.3	36.2 ± 1.1	3.3 ± 0.1	2.2 ± 0.3	12.7 ± 0.5	57.5 ± 1.8	2.8 ± 0.1	7.1 ± 2.4	14.4 ± 0.6	13.0 ± 0.7	66.4 ± 0.5

Cellulose and matrix polysaccharide contents for the 20 lignin mutants (n = 8) and wild-type (n = 16) (± SD) were expressed as percentage CWR. Matrix polysaccharide content was determined gravimetrically based on a TFA extraction and its composition was expressed as mol%. Xylan branching frequency and the percentage methylation of GlcA were determined by DASH. GlcA: glucuronic acid. Increases and decreases compared to the wild-type are indicated in bold and italics, respectively. * 0.001 <*p* < 0.01; ** *p* < 0.001 (Dunnett-Hsu adjusted *t*-test).

The mass loss during TFA extraction can be used as an estimate of the amount of matrix polysaccharides plus amorphous cellulose (Table 3). The CWR of the wild-type contained on average 36% matrix polysaccharides and amorphous cellulose, which is consistent with previous values [47]. However, mutants in the pathway from *C4H* through *CCR1* had an increase in matrix polysaccharide content. Once more, the largest effects were noted for *c4h-2*, *ccr1-3*, and *ccr1-6*, with 21%, 31%, and 25% increase compared to the wild-type, respectively, whereas it was moderate (between 6% and 11%) for the *c4h-3* mutant and both mutant alleles in *4CL1*, *4CL2*, and *CCoAOMT1*.

The major monomeric sugars in the TFA extract quantified by gas chromatography/mass spectroscopy (GC/MS) were arabinose, xylose, and galactose. In both mutant alleles of *CCR1* and *C4H*, the relative amounts of arabinose and galactose were significantly higher than those of the wild-type, but the relative amount of xylose was significantly lower in both mutant alleles of *CCR1*. Although rhamnose, fucose, mannose, and glucose were present in minor quantities, small, but significant, changes could be observed for several mutants. Notably, the reduced amount of crystalline cellulose measured in *ccr1*, *f5h1*, and *comt* mutants was not accompanied by differences in glucose content in the TFA extract that could have been, partially, attributed to increases in amorphous cellulose, indicating that the *ccr1*, *f5h1*, and *comt* mutants indeed had lower levels of total cellulose.

In addition to the general matrix polysaccharide composition, more detailed information on the branching degree of xylan, as well as on the proportion of methylated glucuronic acid (GlcA) on xylan were established by DNA sequencer Assisted Saccharide analysis in High throughput (DASH). The branching degree of xylans was not affected in any of the lignin mutants, but the degree of xylan GlcA methylation was significantly reduced in both *ccr1* mutants and increased in both mutant alleles of *C4H*, *4CL1*, and *CCoAOMT1* (Table 3).

### Most lignin mutants have an improved saccharification yield

To analyze whether the cell wall modifications in the mutant set affected the saccharification yield, a semi-high throughput protocol for small biomass samples (10 mg) was established. Senesced stems of the lignin mutants and the wild-type were cut into 2-mm pieces and saccharified for 48 hours, both without and with acid pretreatment. In this saccharification protocol, a relatively low amount of enzymes was used, allowing a low cellulose conversion into glucose. In this way, even subtle differences in saccharification yield between mutants and wild-type could be revealed.

**Figure 2:**
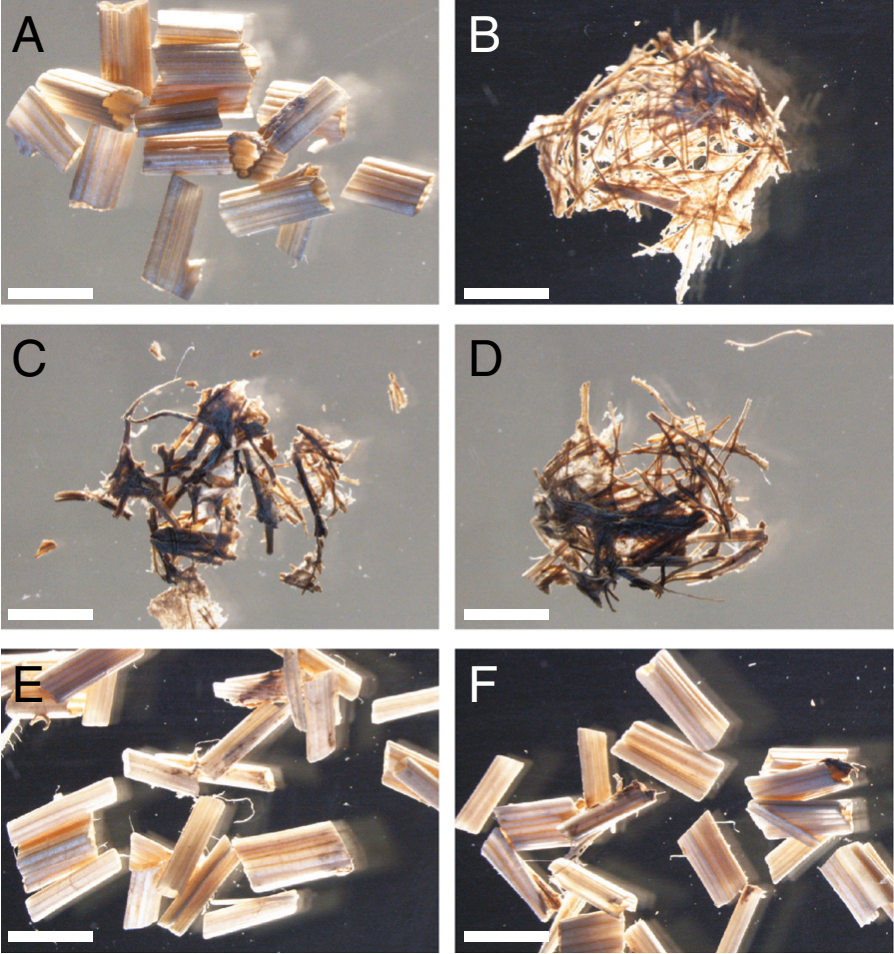
**Cell wall degradation after saccharification.** Stem material after 48 h of saccharification (including acid pretreatment). (**A**) Wild-type. The structure of stem segments of the mutants *c4h-2* (**B**), *ccr1-3* (**C**), and *ccr1-6* (**D**) are fully degraded as a consequence of the almost complete conversion of cellulose into glucose. Although the cellulose conversion was also improved in other mutants, such as *c4h-3* (**E**) and *4cl1-1* (**F**), the stem structure remained intact. Scale bar = 2 mm.

Based on the measured cellulose content (Table 3) and the saccharification yields (Additional file 1), the cellulose conversion was calculated (Table 4). Under our saccharification conditions, approximately 16% and 18% of the cellulose was converted into glucose for the wild-type without and with acid pretreatment, respectively, whereas for the two mutant alleles of *C4H*, *4CL1*, *CCoAOMT1*, *CCR1*, and *COMT,* the cellulose conversions were higher, both without and with acid pretreatment. The *f5h1* mutants had higher cellulose conversions only without pretreatment. Saccharification after acid pretreatment resulted in cellulose conversions that were the highest for *c4h-2*, *ccr1-3*, and *ccr1-6* (approximately 79%, 88%, and 77%, respectively). This almost complete hydrolysis of cellulose in inflorescences of *c4h-2* and *ccr1* mutants during saccharification was also visually noticeable; the structure of the stem segments was completely lost (Figure 2). Although *c4h-3* and the mutant alleles of *4CL1*, *CCoAOMT1*, *F5H1*, and *COMT* also had a cellulose-to-glucose conversion higher than that of the wild-type (albeit lower than that of the *c4h-2* and *ccr1* mutants), their inflorescence stem structure was maintained.

**Table 4:** Cellulose conversions and pretreatment effect

**Line**	**Conversion without (% cellulose)**	**Conversion with (% cellulose)**	**Pretreatment effect (%)**
WT Col-0	16.3 ± 2.2	18.1 ± 2.5	11.2 ± 3.3
*pal1-2*	17.6 ± 2.8	18.5 ± 5.7	5.7 ± 28.0
*pal1-3*	16.8 ± 2.0	19.2 ± 2.4	14.5 ± 3.7
*pal2-2*	17.3 ± 2.6	19.1 ± 3.4	10.3 ± 7.1
*pal2-3*	16.8 ± 1.6	18.1 ± 1.6	8.0 ± 7.0
*c4h-2*	50.5 ± 4.2**	78.9 ± 5.7**	56.4 ± 6.1**
*c4h-3*	24.1 ± 2.6**	41.2 ± 3.7**	71.8 ± 9.7**
*4cl1-1*	21.2 ± 2.1**	30.8 ± 5.1**	44.9 ± 14.7**
*4cl1-2*	20.5 ± 1.9**	29.2 ± 2.8**	43.0 ± 11.4**
*4cl2-1*	18.6 ± 2.5	20.3 ± 2.0	9.8 ± 6.8
*4cl2-3*	17.9 ± 1.0	19.5 ± 1.3	8.9 ± 3.6
*ccoaomt1-3*	21.8 ± 1.2**	29.8 ± 3.3**	36.1 ± 10.4*
*ccoaomt1-5*	20.4 ± 4.6*	33.3 ± 5.4**	75.8 ± 75.3**
*ccr1-3*	77.9 ± 14.6**	88.3 ± 12.8**	14.5 ± 11.1
*ccr1-6*	64.0 ± 4.8**	76.8 ± 5.6**	20.1 ± 6.1
*f5h1-2*	22.8 ± 2.4**	23.1 ± 1.8	1.6 ± 3.3
*f5h1-4*	21.9 ± 2.5**	23.1 ± 2.9	5.8 ± 6.9
*comt-1*	24.8 ± 0.4**	28.5 ± 1.3**	14.9 ± 4.8
*comt-4*	25.4 ± 3.0**	28.2 ± 2.3**	11.4 ± 6.1
*cad6-1*	16.0 ± 1.4	17.9 ± 1.8	11.5 ± 5.2
*cad6-4*	17.8 ± 1.9	20.1 ± 2.7	13.2 ± 5.2

Cellulose conversions for the 20 lignin mutants (n = 8) and the wild-type (WT) (n = 16) (± SD) without and with acid pretreatment were calculated based on the cellulose content and the saccharification yield (both on a CWR basis) and are expressed as percentage cellulose. The pretreatment effect illustrates the percentage increase in cellulose conversion due to the acid pretreatment compared to cellulose conversion without pretreatment and is calculated as follows: (conversion with – conversion without)/(conversion without) × 100 = %. Increases compared to the wild-type are indicated in bold. * 0.001 <*p* < 0.01; ** *p* < 0.001 (Dunnett-Hsu adjusted *t*-test).

The acid pretreatment effect, which is the percentage increase in cellulose conversion due to the pretreatment, was comparable for most lignin mutants with that of the wild-type, i.e., between 2 and 20%. Notable exceptions were the stems of both *c4h*, *4cl1*, and *ccoaomt1* mutants that were more susceptible to the acid pretreatment with a pretreatment effect between 36% and 76%. None of the lignin mutants was more recalcitrant to the acid pretreatment than the wild-type (Table 4).

### Relations between lignin, cell wall polysaccharides, and saccharification yield

**Figure 3:**
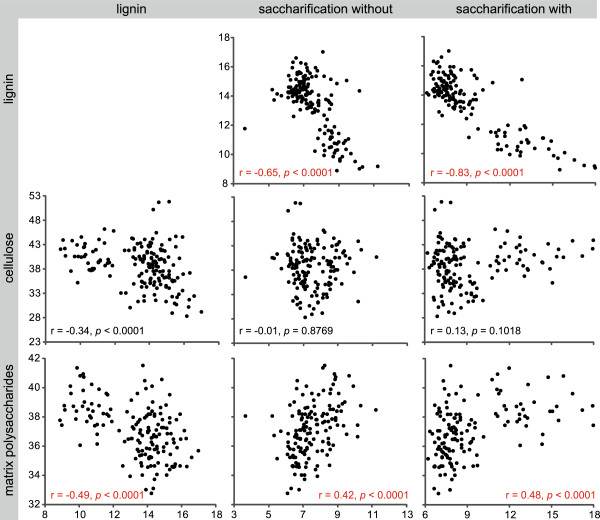
**Interactions between cell wall polymers and saccharification yield.** Scatterplots containing data from the wild-type and all 20 mutants, except *c4h-2*, *ccr1-3*, and *ccr1-6*, illustrating that lignin reduction was compensated for by matrix polysaccharides rather than by cellulose (left column). Scatterplots (middle and right columns), illustrate the relations between saccharification without and with acid pretreatment, respectively, and the different cell wall polymers (lignin, cellulose, and matrix polysaccharides). The Pearson correlation coefficient and its corresponding *p*-value are given at the bottom of each scatterplot and are indicated in red when r > 0.4 and *p* < 0.0001.

To investigate the relations between the different cell wall components and their effect on the saccharification yield without and with acid pretreatment, we calculated the Pearson correlations (Figure 3, Additional file 2, and Additional file 3) based on the compositional data (Tables 2 and 3) and the saccharification data (Table 4 and Additional file 1). However, the data for the three phenotypically abnormal mutants (*c4h-2*, *ccr1-3*, and *ccr1-6*) had too large an effect on the correlations; to minimize the occurrence of high correlations caused by outliers from *c4h-2*, *ccr1-3*, and *ccr1-6* mutants, these three mutants were left out of the analysis. The correlation matrix revealed that a reduction in lignin content was compensated for by an increase in matrix polysaccharides (r = −0.49, *p* < 0.0001) rather than by an increase in cellulose (r = −0.34, *p* < 0.0001), even though the correlation coefficient between lignin and matrix polysaccharides indicated only a weak, and not a strong, relationship. The glucose yields upon saccharification without and with acid pretreatment were correlated (r = 0.80, *p* < 0.0001). Furthermore, the saccharification yield was negatively influenced by the amount of lignin (r = −0.65, *p* < 0.0001 without pretreatment; r = −0.83, *p* < 0.0001 with acid pretreatment). In addition, the data implied that the lignin content played a larger role in determining saccharification yield than the lignin S/G composition (r = −0.65, *p* < 0.0001 and r = 0.31, *p* = 0.0001 for saccharification without pretreatment, respectively; r = −0.83, *p* < 0.0001 and r = 0.59, *p* < 0.0001 for saccharification with pretreatment, respectively). This correlation matrix also revealed that the matrix polysaccharide content influenced the saccharification yield to some extent (r = 0.42, *p* < 0.0001 for saccharification without pretreatment; r = 0.48, *p* < 0.0001 with acid pretreatment), whereas cellulose did not (r = −0.01, *p* = 0.8769 for saccharification without pretreatment; r = 0.13, *p* = 0.1018 for saccharification with acid pretreatment).

Next, models were made that described how the different cell wall polymers influenced the saccharification yield (Table 5 and Additional file 4). By modeling the saccharification yield (Additional file 1) and not the cellulose conversion (Table 4), the cell wall parameter “crystalline cellulose content” could be included into the model. The other cell wall parameters that were considered to influence the saccharification yield and, hence, design the saccharification model were the amount of lignin and matrix polysaccharides, the S/G ratio, the abundance of ferulic acid, and the three main matrix monosaccharides arabinose, galactose, and xylose. For the three monosaccharides, the absolute amounts (Additional file 5) were used to build the models instead of the molar proportions (Table 3), because the relative proportions for these sugars are intrinsically linked with each other. Generally, the models confirmed the correlation matrix and explained 70% and 71% of the saccharification yield variations without and with acid pretreatment, respectively (Table 5). When saccharification was performed without pretreatment, the lignin amount had the strongest negative impact on the saccharification yield, followed by the S/G ratio, xylose, and ferulic acid content. Galactose and the amount of matrix polysaccharides contributed positively to the saccharification yield without pretreatment. Similarly, for saccharification with acid pretreatment, the lignin content and xylose had a negative impact and only galactose, but not the other matrix polysaccharides sugars or content, positively influenced the saccharification yield with acid pretreatment.

**Table 5:** Models for saccharification yield and pretreatment effect

**Cell wall factor**	**Parameter estimate**	**Standard error**	***p*-value**	**Standardized estimate**
***Saccharification model without pretreatment***				
Intercept	0.09536	0.01308	<0.0001	0.00
Lignin	−0.52154	0.04720	<0.0001	−0.89
S/G	−0.01170	0.00318	0.0003	−0.29
Ferulic acid	−4.24820	1.27867	0.0011	−0.15
Galactose	0.00223	0.00066	0.0009	0.24
Xylose	−0.00044	0.00015	0.0043	−0.19
Matrix polysaccharides	0.07289	0.03156	0.0224	0.13
***Saccharification model with acid pretreatment***				
Intercept	0.52417	0.04977	<0.0001	0.00
Lignin	−8.14968	0.89507	<0.0001	−5.33
Lignin^2^	35.18722	4.45747	<0.0001	4.61
Galactose	0.00538	0.00149	0.0004	0.22
Xylose	−0.00120	0.00036	0.0010	−0.20
***Pretreatment effect model***				
Intercept	0.29129	0.04709	<0.0001	0.00
Lignin	−5.35810	0.79704	<0.0001	−4.91
Lignin^2^	24.30648	3.91171	<0.0001	4.46
S/G	0.01737	0.00693	0.0133	0.23
Galactose	0.00384	0.00144	0.0087	0.22
Xylose	−0.00091	0.00033	0.0058	−0.21

Models illustrate which cell wall factors had a significant influence on the saccharification yield or pretreatment effect. The “parameter estimate” indicates the increase in saccharification yield or pretreatment effect when 1 unit of the cell wall factor is increased when the other cell wall factors are fixed. The standardized estimate indicates the strength of the influence on the saccharification yield or pretreatment effect. Lignin^2^ = lignin x lignin. All data used to build these models can be found in Additional file 4.

In addition, the increase in saccharification yield that was attributable to the acid pretreatment, represented as the “pretreatment effect”, was also negatively influenced by the lignin content and xylose, but positively by the S/G ratio and galactose (Table 5). Because arabinose was correlated with galactose (r = 0.94, *p* < 0.001), arabinose was left out of the model, but it would influence the saccharification yield and pretreatment effect in a similar way as galactose. Strikingly, cellulose content did not affect the saccharification yield, neither without, nor with pretreatment, and did not influence the pretreatment effect.

## Discussion

Two mutant alleles for 10 genes of the phenylpropanoid and monolignol biosynthetic pathways were grown together with the wild-type and their senesced inflorescences analyzed for cell wall composition (lignin content, lignin S/G composition, nonconventional lignin units, crystalline cellulose content, and matrix polysaccharide content and composition) and saccharification yields without and with acid pretreatment. The high number of mutants analyzed allowed us to make system-wide correlations among the various parameters. In addition, we proposed saccharification models in which the saccharification yield was considered as a function of the cell wall composition that identified the major cell wall recalcitrance-determining factors.

### System-wide effects on lignin

Although the lignin biosynthetic pathway has been studied extensively by means of reverse and forward genetics [48], the systematic side-by-side comparison of the 20 mutants, each defective in a single gene of the lignin biosynthetic pathway, revealed a number of novel observations. First, the *c4h*, *4cl1*, *ccoaomt1*, and *ccr1* mutants had a reduced lignin content, but not *4cl2*, probably because *4CL2* has a 10-fold lower substrate specificity for 4-coumarate than *4CL1*[49]. The *pal1* and *pal2* mutants had normal lignin levels, presumably because of gene redundancy. Indeed, the *pal1 pal2 pal3 pal4* quadruple mutant had only 20-25% of residual lignin [50]. As *F5H1*, *COMT*, and *CAD* are specific for the biosynthesis of S units, disruption of these genes has a larger impact on the lignin composition than on the lignin content. Although lignin is a load-bearing polymer in the secondary cell wall, most lignin mutants had no obvious morphological phenotypes, despite a reduced lignin content. For example, the *4cl1* mutants maintained a normal phenotype with 26% reduced lignin levels and even the *c4h-3* mutant with a lignin content of only 64% of that of the wild-type grew normally under our growth conditions.

Second, the lignin composition as analyzed via thioacidolysis clearly differed in mutants of the general phenylpropanoid pathway (*C4H*, *4CL1*, and *CCoAOMT1* with an increase in the S/G ratio) and the monolignol-specific pathway (from *CCR1* through *CAD6* with a decrease in the S/G ratio). Because *F5H1* and *COMT* are specific for S biosynthesis, it is not surprising that the *f5h1* and *comt* mutants lack S units. Given that S biosynthesis increases during stem development [51], the decrease in S units in the *ccr1* mutants can be attributed to their slower and probably incomplete development [34,52,53]. The reduction in the S unit content can easily be explained for the *ccr1*, *f5h1*, and *comt* mutants, but the increase in S units in the *c4h* and *4cl1* mutants is more difficult to explain from the linear pathway presented in Figure 1. Most likely, the residual flux through the phenylpropanoid pathway preferentially proceeds toward S units when F5H1 and COMT remain fully active, because F5H1 is known to be the rate-limiting step in sinapyl alcohol biosynthesis [54]. Alternatively, in the mutants, feed-back and feed-forward mechanisms can alter the flux through the different steps of the lignin biosynthetic pathway [32,55].

Third, both mutant alleles of *CCR1* had a relative increase in thioacidolysis-released H units. The accumulation of H units in the *ccr1* mutants is puzzling because the corresponding enzyme is positioned before the biosynthesis of H units; however, microarray data of inflorescence stems of the *ccr1* mutants revealed that the transcript level of *CCR2* (and not of *CCR1*) was higher than that of the wild-type [32], and might contribute to the formation of H units [56]. Hence, a possible redirection of the pathway could be that the CCR2 activity takes the flux partially to H units in the *ccr1* mutants. The potential involvement of *CCR2* in the production of H units has recently been suggested in alfalfa as well [55]. Nevertheless, additional enzyme kinetics and flux studies are needed to demonstrate these alternative pathways.

Fourth, compounds derived from an incomplete monolignol biosynthesis often incorporate into the lignin polymer of lignin biosynthesis mutants [57]. In line with previous reports, 5-OH-G units, FA-derived units, and S aldehyde units were evidenced in lignin of *COMT*-deficient [31], *CCR*-deficient [43,44], and *CAD*-deficient plants [40-42], respectively. Analysis of the lignin composition of the entire set of lignin mutants revealed that these nonconventional lignin units are specific for the mutants described above and do not occur at high levels in the other lignin mutants analyzed.

### Relations between lignin and cell wall polysaccharides

Perturbations in the lignin biosynthetic pathway have been shown to have far-reaching consequences on the transcriptome and metabolome [32,55,58-61]. The scientific literature often suggests that reductions in lignin amount are compensated for by increases in cellulose content [29,31]. In contrast, we did not observe such a compensation in *Arabidopsis*. None of the mutants displayed an increase in cellulose content at senescence or in cellulose synthase (*CesA*) transcript levels, as analyzed by microarrays [32]. Instead, the reduction in lignin levels in the *c4h*, *4cl1*, *ccoaomt1*, and *ccr1* mutants was rather associated with an increase in matrix polysaccharide levels, according to the weight loss after a TFA extraction (Figure 3, Additional file 2, and Additional file 3). Besides an effect on the matrix polysaccharide content, its composition was also affected by the mutations in the lignin biosynthetic pathway. The differences were the largest in the *c4h* and *ccr1* mutants, although a large proportion of these differences in the *ccr1* mutants could potentially be due to the altered development rather than to a compensation mechanism. Although in some lignin mutants the matrix polysaccharide composition was slightly altered, no obvious correlations were found between lignin content or composition and matrix polysaccharide composition. However, whereas all mutants had similar levels of branching xylans, the *c4h*, *4cl1*, and *ccoaomt1* mutants had strikingly more and the *ccr1* mutants less methylation of GlcA. Previously, increases in GlcA methylation on xylan had been observed only in xylan biosynthesis mutants [6]. However, more in-depth studies are needed to fully understand how the lignin and matrix polysaccharide pathways are interconnected.

Another remarkable observation was the decreased cellulose content in the *ccr1*, *f5h1*, and *comt* mutants. The low cellulose amount in both *ccr1* mutants might possibly be due to their altered development [34,52,53,62], but the *f5h1* and *comt* mutants developed normally, albeit with reduced cellulose contents. Common to all mutants with a decreased cellulose content (*ccr1*, *f5h1*, and *comt*) is the release of fewer S units upon thioacidolysis and, consequently, a decreased S/G ratio and a high lignin condensation (H + G + S) (Table 2 and Additional file 6). Mutants with increased S content and S/G ratio (*c4h*, *4cl1*, and *ccoaomt1*) had a cellulose content similar to that of the wild-type. These data suggest that when the S/G ratio drops below a certain level, the crystalline cellulose content in the cell wall decreases. In contrast, when the S/G ratio is elevated, at least ranging from 0.41 in the wild-type up to 0.86 in the *c4h-3* mutant (Table 2), the crystalline cellulose content remained equal to that of the wild-type. The positive correlation between lignin composition (S/G) and cellulose also follows from the Pearson correlation coefficient in the correlation matrix (r = 0.57, *p* < 0.0001) (Additional file 3). A correlation between the S/G ratio and cellulose had also been found recently in eucalyptus by studying natural variation in wood properties [63]. These observations raise the question as whether monolignol biosynthesis affects cellulose deposition directly or indirectly, especially because lignin is supposed to be deposited mainly in the secondary cell wall *after* completion of the cellulose biosynthesis [7].

### Lignin content, lignin composition, galactose, xylose, and matrix polysaccharide content, but not cellulose content, affect saccharification yield

Based on the lignin content and saccharification yield of the different mutants (Tables 2 and 4) and the correlation matrix (Figure 3, Additional file 2, and Additional file 3), it is clear that saccharification yield is highly influenced by the lignin content, as also shown in alfalfa [3]. However, in both mutant alleles of *COMT*, an increase in the saccharification yield occurred without and with acid pretreatment, but no decrease in lignin content. These exceptions indicate that other cell wall parameters, besides lignin content, influence saccharification.

Saccharification of the *f5h1* mutants (low S/G) and overexpression of the *Arabidopsis F5H1* gene (high S/G) revealed that the lignin composition (via the traditional monomers) had no influence on the saccharification yield without pretreatment, but high S lignins had an improved saccharification after pretreatment with hot water [23]. Similar observations with a hot water pretreatment were found for saccharification of wood from natural poplar variants [19]. In contrast, in our saccharification models, which are based on a range of S/G ratios and not on extreme S/G ratios only and use a pretreatment different from hot water, the S/G ratio had a negative effect when no pretreatment was included, but not when an acid pretreatment preceded the saccharification (Table 5). This suggests that cell walls with a high S/G ratio, form a matrix in which the matrix polysaccharides (that are the targets for acid pretreatment) render the cellulose less accessible by cellulases.

The saccharification models clearly revealed that the lignin content was the main factor determining saccharification, whether a pretreatment was included or not. When saccharification was carried out without a pretreatment, the S/G lignin composition, ferulic acid content, and xylose content also negatively influenced the saccharification yield, whereas matrix polysaccharide content and galactose had a positive effect. The saccharification yield with acid pretreatment was only influenced negatively by the lignin and xylose contents and positively by the galactose content. Because arabinose was positively correlated with galactose, it would influence saccharification yield in a similar way as galactose. For example, a 10% increase in saccharification yield with pretreatment, as compared to wild-type (i.e., from 0.059 to 0.065 mg/mg dry weight), can be obtained by a lignin reduction of 9.9% (i.e., from 0.114 to 0.103 mg/mg dry weight). A similar increase in saccharification yield could also be achieved by increasing the galactose content by 8.5% (i.e., from 12.9 to 14.0 μg/mg dry weight), increasing the arabinose content by 12% (i.e., from 11.0 to 12.4 μg/mg dry weight), or decreasing the xylose content by 9.5% (i.e., from 52.1 to 47.2 μg/mg dry weight). Remarkably, without or with acid pretreatment, the cellulose content was not important for the saccharification yield. However, only 70% and 71% of the variation in saccharification yield without and with pretreatment, respectively, could be explained by these saccharification models, indicating that factors other than the ones examined here might still play a role in cell wall recalcitrance. Importantly, it is intrinsic to models that they only predict the outcome within the range of the data. Care must thus be taken by extrapolating the predicted effects beyond that range.

## Conclusions

Two mutant alleles for 10 genes of the phenylpropanoid and monolignol biosynthetic pathways were grown together with the wild-type and analyzed for their cell wall composition and saccharification yield. Our data suggest that, at least in *Arabidopsis*, the reduction in lignin is not compensated for by an increase in cellulose, but rather by an increase in matrix polysaccharides. The mutants *c4h-2*, *ccr1-3*, and *ccr1-6*, with the largest reduction in lignin content, had the highest saccharification yields and an almost complete cellulose conversion that resulted in a stem structure disintegration. The saccharification models indicated that lignin content was the main factor determining saccharification yield. Without pretreatment, also lignin composition played a role, whereas with acid pretreatment lignin composition was not important anymore. In both cases, without and with acid pretreatment, other cell wall factors, such as xylose, galactose, and arabinose contents, affected the saccharification yields. Our results contribute to a better understanding of the effect of lignin perturbations on plant cell wall composition and its influence on saccharification yield. These results provide new potential targets for genetic improvement, such as the biosynthesis of arabinogalactan, mannans, or xyloglucans to increase the galactose content.

## Methods

### Plant material

For 10 different genes involved in lignin biosynthesis, two *Arabidopsis thaliana* (L.) Heyhn. mutant alleles were used in this study, including *pal1-2*, *pal1-3*, *pal2-2*, *pal2-3*, *c4h-2*, *c4h-3*, *4cl1-1*, *4cl1-2*, *4cl2-1*, *4cl2-3*, *ccoaomt1-3*, *ccoaomt1-5*, *ccr1-3*, *ccr1-6*, *f5h1-2*, *f5h1-4*, *comt-1*, *comt-4*, *cad6-1*, and *cad6-4*. For a schematic presentation of the 20 mutants and their residual expression, see Vanholme *et al*. [32]. Sixteen biological replicates of each homozygous mutant and 32 biological replicates for the wild-type were grown simultaneously in a random block design, spread over different trays, in the same environment. Because of their delayed development, *c4h-2*, *ccr1-3*, and *ccr1-6* were planted 2 weeks in advance to allow simultaneous bolting. Plants were grown first under short-day conditions (8 h light, 21°C, and 55% humidity) during 6 weeks and then transferred to long-day conditions (16 h light, 21°C, and 55% humidity) to allow the development of a single tall inflorescent stem. For all biological repeats, the main stem was harvested just above the rosette when the plant was completely senesced and dry. Once harvested, rosette leaves, axillary inflorescences, and siliques were removed. The main stem was weighed and the bottom 1 cm was removed. The lowest 10 cm of the remaining stem was chopped in 2-mm pieces. Biological repeats were pooled two by two to obtain 8 biological replicates for the mutant alleles and 16 repeats for the wild-type. These pooled samples were used for wet-chemistry cell wall analyses and saccharification assays.

### Lignin analyses

Aliquots of 5 mg stem pieces were subjected to a sequential extraction to obtain a purified CWR. The extractions were done in 2-ml vials, each time for 30 min, at near boiling temperatures for water (98°C), ethanol (76°C), chloroform (59°C), and acetone (54°C). The remaining CWR was dried under vacuum. Lignin was quantified according to a modified version of the acetyl bromide method [35], optimized for small amounts of plant tissue. The dried CWR was dissolved in 0.1 ml freshly made 25% acetyl bromide in glacial acetic acid and 4 μl 60% perchloric acid. The solution was incubated for 30 min at 70°C while shaking (850 rpm). After incubation, the slurry was centrifuged at 23,477 *g* for 15 min. To the supernatant, 0.2 ml of 2 M sodium hydroxide and 0.5 ml glacial acetic acid were added. The pellet was washed with 0.5 ml glacial acetic acid. The supernatant and the washing phase were combined and the final volume was adjusted to 2 ml with glacial acetic acid. After 20 min at room temperature, the absorbance at 280 nm was measured with a NanoDrop® ND-1000 spectrophotometer (Thermo Scientific, Wilmington, DE, USA). The lignin concentrations were calculated by means of the Bouguer-Lambert-Beer law: A = ϵ × l × c, with ϵ = 23.35 l g^-1^ cm^-1^[64] and l = 0.1 cm.

The lignin composition was investigated with thioacidolysis as previously described [65]. The monomers involved in β–O–4-ether bonds, released upon thioacidolysis, were detected with gas chromatography (GC) as their trimethylsilyl (TMS) ether derivatives on a Hewlett-Packard HP 6890 Series GC system (Agilent, Santa Clara, CA, USA) coupled with a HP-5973 mass-selective detector. The GC conditions were as described [65]. The quantitative evaluation was carried out based on the specific prominent ions for each compound. A summary of the specific ions for each identified compound can be found in Additional file 7. Response factors for H, G, and S units were taken from [66]. Because we had no standards for the minor lignin units, a response factor of 0.47 was used, which is the average of the three response factors for the major lignin units.

### Polysaccharide analyses

Aliquots of 4 mg dry stem pieces were sequentially extracted to obtain a purified CWR, as described above. To estimate the amount of cellulose, we used a colorimetric method (based on [45,46]). The CWR was incubated with 2 M TFA and 20 μl inositol (5 mg ml^-1^) for 2 h at 99°C while shaking (750 rpm). This TFA extract was used for the determination of the sugar composition of matrix polysaccharides (see below). After incubation, the remaining pellet was washed three times with water and twice with acetone and dried under vacuum. Concentrated sulfuric acid (150 μl) and 30 μl 5% (w/v) phenol (freshly made in water) were added to the dried pellet and incubated for 1 h at 90°C with gentle shaking (500 rpm). After centrifugation for 3 min at 23,477 *g*, a 50 μl aliquot of the supernatant was diluted 20 times with MilliQ water (Millipore, Billerica, MA, USA) to measure the absorbance at 493 nm. The amount of cellulose was calculated back from a standard curve of Avicel® PH-101 (FMC BioPolymer, Philadelphia, PA, USA).

To determine the different monosaccharides present in the TFA extract, 800 μl TFA extract was dried under vacuum and further converted to the corresponding alditol acetates as described [67]. The GC-MS analyses were carried out with a mass-selective detector (HP 5973 model; Agilent), interfaced to a GC (HP 6890 model; Agilent) equipped with an automated sample injector and an VF-5 ms capillary column (30 m × 0.25 mm). The GC conditions were as described [68]: the oven was kept at 100°C for 1 min, increasing the temperature to 245°C at a rate of 20°C min^-1^, held at 245°C for 30 min, and decreasing the temperature to a final temperature of 100°C at a rate of 30°C min^-1^. Peak areas of the different sugars were normalized with the peak area of the internal standard inositol (20 μl, 5 mg ml^-1^). Response factors were determined based on standard curves for each of the different sugars: rhamnose (2.01), fucose (2.05), arabinose (1.35), xylose (1.35), mannose (1.45), glucose (1.59), and galactose (1.55).

### Analysis of xylan structure

Senesced stems (10 mg) from five biological replicates of each genotype were incubated at 70°C in 96% ethanol for 20 min and then homogenized using a ball mixer mill (Glen Creston, London, UK). The insoluble material was washed with 100% ethanol, twice with chloroform:methanol (2:1), and then successively washed with 65%, 80%, and 100% ethanol prior to air drying to produce an alcohol-insoluble residue (AIR). AIR (100 μg) was pretreated with 4 M NaOH (20 μl) for 1 h at 21°C to make the xylan enzyme accessible, neutralized, and then resuspended in 500 μl 0.1 M ammonium acetate buffer (pH 5.5). Samples were incubated overnight at 21°C with an excess of the xylanase NpXyn11A (a kind gift of Harry Gilbert, University of Newcastle, UK) to ensure complete digestion.

The samples were dried under vacuum and analyzed by DNA sequencer Assisted Saccharide analysis in High throughput (DASH). The xylanase-released oligosaccharides or quantitation standards and appropriate controls were derivatized with 8-aminopyrene-1,3,6-trisulfonic acid (APTS; Biotium, Hayward, CA, USA). The dried oligosaccharide sample was combined with 10 μl APTS (0.02 M in 1.2 M citric acid) and 10 μl 0.1 M NaCNBH_3_ solutions. Following overnight incubation (30°C), the samples were diluted to 1 μg ml^-1^ initial AIR, of which 10 to 30 μl were loaded into a 96-well microtiter plate, and analyzed by capillary electrophoresis with a laser-induced fluorescence (CE-LIF) on an Applied Biosystems 3730xl DNA Analyzer (Life Technologies, Carlsbad, CA, USA). Peaks were identified by co-migration with known standards and quantified based on peak area using quantitation standards analyzed in parallel. Oligosaccharides Xyl, Xyl_2_, GlcA Xyl_4_, and MeGlcA Xyl_4_ were used to calculate the degree of xylan branching and the proportion of GlcA methylation, as described previously with the polysaccharide analysis using the carbohydrate gel electrophoresis technique [69].

### Saccharification assays

The protocol for saccharification of senesced *Arabidopsis* inflorescences was as follows. Aliquots of 10 mg of dry 2-mm stem segments were used. The biomass was pretreated with 1 ml of 1 N HCl at 80°C for 2 h, while shaking (850 rpm). The acid extract was removed and the pretreated material was washed three times with 1 ml water to obtain neutral pH. Subsequently, the material was incubated with 1 ml 70% (v/v) ethanol overnight at 55°C. The remaining biomass was washed three times with 1 ml 70% (v/v) ethanol, once with 1 ml acetone, and dried under vacuum for 45 min. For the saccharifications without pretreatment, 10 mg aliquots of dry stem segments were immediately incubated with 1 ml 70% (v/v) ethanol overnight at 55°C. The extracted material was washed three times with 1 ml 70% (v/v) ethanol, once with 1 ml acetone, dried under vacuum for 45 min, and weighed. As this overnight ethanol extraction was an alternative procedure for cell wall preparation, the percentage of CWR in dry matter, specific for saccharification, could be calculated based on the weights before and after overnight ethanol extraction. As inclusion of the acid pretreatment prior to the overnight ethanol extraction removed some cell wall components, weighing the material after acid pretreatment and ethanol extraction overnight underestimated the amount of CWR. Therefore, the CWR data from the untreated samples were taken to calculate the glucose release with acid pretreatment per CWR.

The ethanol-extracted residue, after acid pretreatment or no pretreatment, was dissolved in 1 ml acetic acid buffer solution (pH 4.8) and incubated at 50°C. The enzyme mix added to the dissolved material contained cellulase from *Trichoderma reseei* ATCC 26921 and β-glucosidase (Novozyme, Bagsvaerd, Denmark) in a 5:3 ratio. Both enzymes were first desalted over an Econo-Pac 10DG column (Bio-Rad, Hercules, CA, USA), stacked with Bio-gel® P-6DG gel (Bio-Rad) according to the manufacturer’s guidelines. The desalted β-glucosidase was 350-fold diluted prior to mixing with desalted cellulase. The enzyme mix was further diluted 10-fold and the activity of the diluted enzyme mix was measured with a filter paper assay [70]. To each biological sample, dissolved in acetic acid buffer (pH 4.8), the enzyme mix with an activity of 0.06 filter paper units was added. After a short spinning to remove droplets from the lid of the reaction tubes, 20 μl aliquots of the supernatant were taken after 48 h of incubation at 50°C and 30-fold diluted with acetic acid buffer (pH 4.8). The concentration of glucose in these diluted samples was measured indirectly with a spectrophotometric color reaction (glucose oxidase-peroxidase; GOD-POD). A 100 ml aliquot of the reaction mix from this color reaction contained 50 mg 2,2′-azino-bis(3-ethylbenzthiazoline-6-sulfonic acid), 44.83 mg GOD (Sigma-Aldrich, St. Louis, MO, USA), and 173 μl of 4% (w/v) POD (Roche Diagnostics, Brussels, Belgium) in acetic acid buffer (pH 4.5). To measure the glucose concentration, 50 μl of the diluted samples was added to 150 μl GOD-POD solution and incubated for 30 min at 37°C. The absorbance was measured spectrophotometrically at a wavelength of 405 nm. The concentration in the original sample was calculated with a standard curve based on known d-glucose concentrations (Sigma-Aldrich).

### Descriptive statistics and significance tests

All statistical analyses were performed with SAS® 9.2 (SAS Institute Inc., 2008, Cary, North Carolina). Mixed model analysis was performed for each variable to test whether there was a significant line effect. Tray was put as a random effect in the model. The significance of the tray effect was assessed with a likelihood ratio test, using a mixture distribution of χ_1_^2^ and χ_0_^2^. When model assumptions were not met, box-cox powertransformations were applied to ensure the validity of the model. All variables were ≥0. To variables that also contained values between 0 and 1, the value 1 was added prior to transformation. Osborne [71] pointed out that for some transformations numbers between 0 and 1 are treated differently than numbers above 1. In cases where no suitable powertransformation was possible, the nonparametric Friedman test was used. The applied transformations were as follows:

H = square root of (H+1);

*bis*-β–O–4-FA = (*bis*-β–O–4-FA +1)^0.2^;

β–O–4-FA = log_e_ (β–O–4-FA +1);

G aldehyde = 1/(Gald+1);

S aldehyde = 1/(Sald+1);

glucose = 1/square root (glucose);

galactose= log_e_ (galactose)

A nonparametric analysis was applied to % H, % G, % S. Post-hoc Dunnett’s tests (2-sided) were performed to test for significant differences between a particular line and wild-type. Differences with a Dunnett adjusted *p*-values < 0.01 were considered significant. Differences are reported on their original scale, however the null hypotheses are valid on the transformed scale only. For the nonparametric test approximations to the rank-sum multiple comparisons were obtained.

Saccharification yield (mg glucose/mg CWR) was measured at different timepoints. A repeated measurements analysis was performed using a linear spline model with knots at timepoints 3 h, 8 h and 24 h based on the line plots. Several covariance structures were modelled. The model with the lowest AIC value was the model with an unstructured covariance. Tray was also included in the model as a random effect. The significance of the tray effect was assessed with a likelihood ratio test, using a mixture distribution of χ_1_^2^ and χ_0_^2^. The full model was:

Saccharification yield = intercept+tray+line+time+time*line+time3+time3*line+time8+time8*line+time24+time24*line.

One-sided post-hoc Dunnett’s tests were performed at 48h to test for significant increases in saccharification yield in a particular line compared to wild-type. Separate analyses were performed for the experiments with and without pretreatment.

### Statistical modeling of saccharification yield

To understand which factors had the most significant influence, the saccharification yield in mg per mg dry weight at 48 h was modeled separately for the analysis with and without pretreatment by means of multiple linear regression. Data from the two *ccr1* and the *c4h-2* mutants were not taken into account. The factors that were considered to influence the glucose release were lignin (mg), S/G, ferulic acid (*bis*-β–O–4-FA, μmol), the three most abundant sugars (arabinose (μg), galactose (μg), and xylose (μg)), cellulose (mg), and matrix polysaccharides (mg). All factors were expressed on a mg dry weight basis. In a first step, all correlations between the different independent variables were investigated to avoid multicollinearity. As the correlation between arabinose and galactose (r = 0.94, *p* < .0001) was very high, arabinose was eliminated from the model. Multicollinearity was monitored with the variance inflation factor (VIF) of which the square root indicates how much larger the standard error is compared with what it would be if the variables were uncorrelated with the other independent variables in the equation. Models were built by manual backward selection with removal of one severe outlier. The R^2^ of the final model for the saccharification yield was 0.70 and 0.71 without and with pretreatment, respectively. The effect of pretreatment was also modeled with the same covariates; the R^2^ of the final model for this pretreatment effect was 0.58.

## Abbreviations

4CL: 4-coumarate:CoA ligase; 5-OH-G: 5-hydroxyguaiacyl; AcBr: Acetyl Bromide; AIR: Alcohol-insoluble residue; C3H: *p*-coumarate 3-hydroxylase; C4H: Cinnamate 4-hydroxylase; CAD: Cinnamyl alcohol dehydrogenase; CCoAOMT: caffeoyl-CoA *O*-methyltransferase; CESA: Cellulose synthase; COMT: Caffeic acid *O*-methyltransferase; CCR: Cinnamoyl-CoA reductase; CWR: Cell wall residue; DASH: DNA sequencer Assisted Saccharide analysis in High throughput; F5H: Ferulate 5-hydroxylase; FA: Ferulic acid; G: Guaiacyl; GlcA: Glucuronic acid; GOD: Glucose oxidase; H: *p*-hydroxyphenyl; HCT: *p*-hydroxycinnamoyl-CoA:quinate shikimate *p*-hydroxycinnamoyltransferase; PAL: Phenylalanine ammonia lyase; POD: Peroxidase; S: Syringyl; TFA: Trifluoroacetic acid; TMS: Trimethylsilyl; VIF: Variance inflation factor.

## Competing interests

The authors declare that they have no competing interests.

## Authors’ contributions

RVA and WB designed this study. RVA grew plants, carried out phenotypic analyses, cell wall characterizations, and saccharification assays. VS built the saccharification models and performed statistical analysis. JM performed DASH. PD helped interpreting the DASH data. RVA, RV, and WB wrote the manuscript. All authors read and approved the final manuscript.

## Supplementary Material

Additional file 1 **Saccharification yields for the 20 mutants (n = 8) and wild-type (n = 16) (± SD) without and with acid pretreatment.** Saccharification yields are expressed as percentage CWR. Increases and decreases compared to the wild-type are indicated in bold and italics, respectively. * 0.001 < *p* < 0.01; ** *p* < 0.001 (Dunnett-Hsu adjusted *t*-test). Click here for file

Additional file 2 **Scatterplots containing data from the wild-type and all 20 mutants, except *c4h-2*, *ccr1-3*, and *ccr1-6*, illustrating that lignin reduction was compensated for by matrix polysaccharides rather than by cellulose (left column).** The distinction between the different mutants and the wild-type is visualized by different colors. Scatterplots (middle and right columns) illustrate the relations between saccharification without and with acid pretreatment, respectively, and the different cell wall polymers (lignin, cellulose, and matrix polysaccharides). The Pearson correlation coefficient and its corresponding *p*-value are given in the upper left corner of each scatterplot. Click here for file

Additional file 3 **Pearson correlation matrix between phenotypic traits, cell wall polymers, and saccharification.** The data of the wild-type and all mutants, except *c4h-2*, *ccr1-3*, and *ccr1-6*, were used to build the correlation matrix (Tables 1 to 4 and Additional file 1). The top half gives the *p*-values, the bottom half the Pearson correlation coefficients. Orange: Pearson correlation coefficient (r) (and their corresponding *p*-values) >0.7; blue: 0.6 < r < 0.7; green: 0.5 < r < 0.6. A correlation coefficient of 0.7 indicates a strong linear relation, of 0.6 < r < 0.7, a medium strong relation, and of 0.5 < r < 0.6, a weak linear relation. Sacch 0: saccharification yield without pretreatment; sacch 1: saccharification yield with acid pretreatment; conversion 0: cellulose conversion without pretreatment; conversion 1: cellulose conversion with acid pretreatment. Click here for file

Additional file 4 **Data used to build the models for saccharification yield without and with acid pretreatment and pretreatment effect.** Saccharification yield without pretreatment (sacch0), with pretreatment (sacch1), lignin, cellulose, and matrix polysaccharides are expressed as mg per mg dry weight. Ferulic acid is expressed as μmol per mg dry weight. Arabinose, xylose, and galactose are expressed as μg mg^-1^ dry weight. Click here for file

Additional file 5 **Matrix polysaccharide composition for the 20 lignin mutants (n = 8) and wild-type (n = 16) (± SD).** The absolute amounts of the different sugars are expressed as μg mg^-1^ dry weight. These absolute values for arabinose, xylose, and galactose were used to build the saccharification models. Increases compared to the wild-type are indicated in bold. * 0.001 < *p* < 0.01; ** *p* < 0.001 (Dunnett-Hsu adjusted *t*-test). Click here for file

Additional file 6 **Lignin composition for the 20 lignin mutants (n = 8) and wild-type (n = 16) (± SD).** The absolute amounts of the different lignin monomers detected by thioacidolysis are expressed as μmol g^-1^ AcBr lignin. Increases and decreases compared to the wild-type are indicated in bold and italics, respectively. * 0.001 <*p* < 0.01; ** *p* < 0.001 (Dunnett-Hsu adjusted *t*-test). Click here for file

Additional file 7 **List of specific prominent ions used to extract the ion-specific chromatograms and quantify the different lignin units, released during thioacidolysis, in the 20 mutants and the wild-type.** Target ions and qualifiers are *m/z* values. Click here for file
