# Supplementary figures and images for: Lignin biosynthesis perturbations affect secondary cell wall composition and saccharification yield in Arabidopsis thaliana
Source: Biotechnol Biofuels. 2013 Apr 26;6:46. doi: 10.1186/1754-6834-6-46 (PMC3661393; doi:10.1186/1754-6834-6-46)

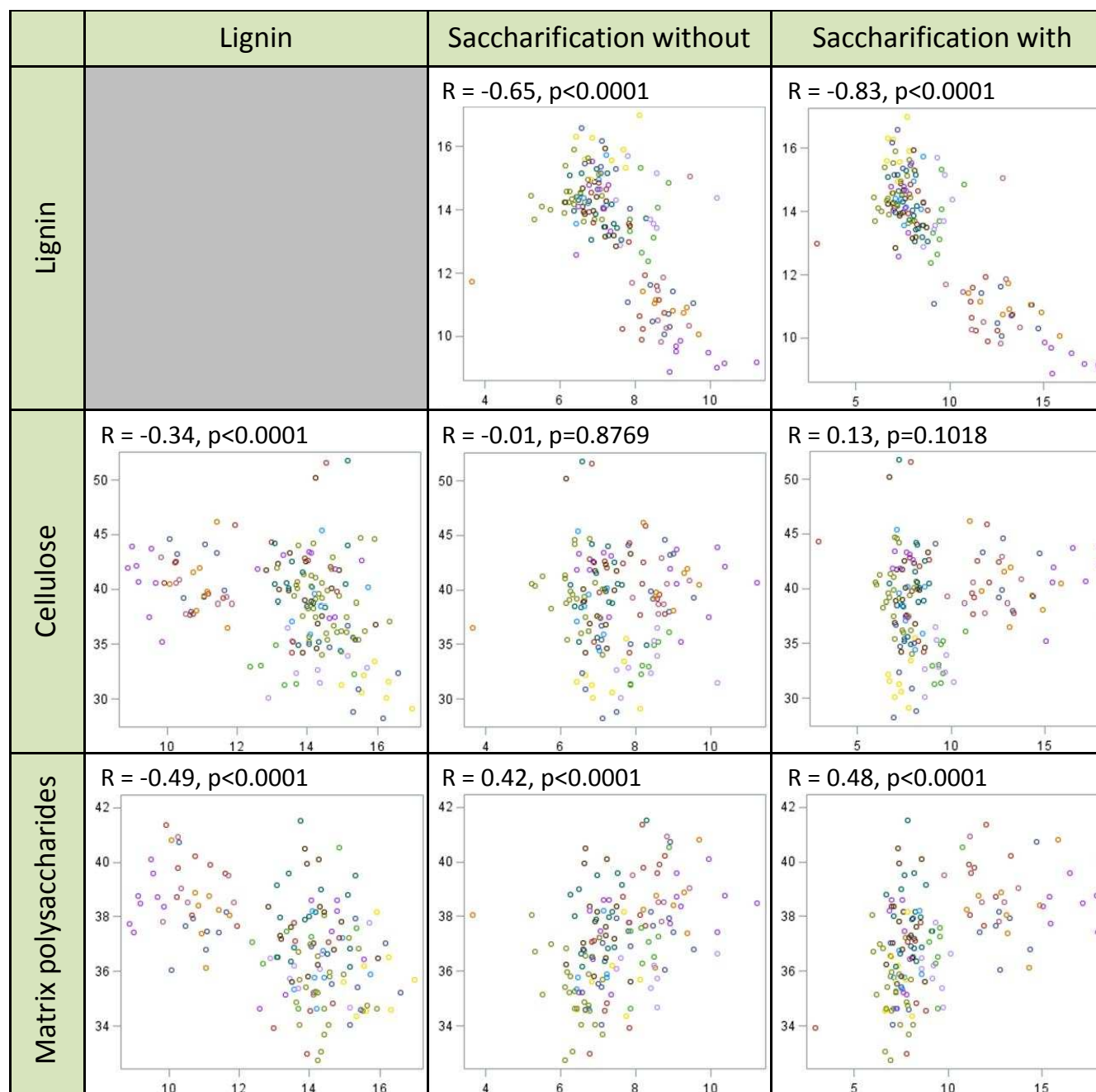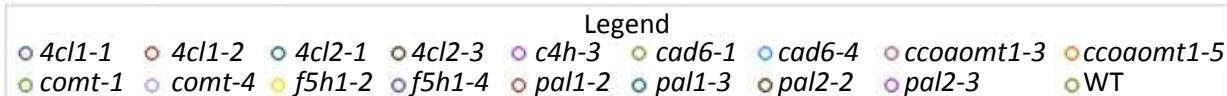

Supplement: Additional file 2 — Scatterplots containing data from the wild-type and all 20 mutants, except c4h-2, ccr1-3, and ccr1-6, illustrating that lignin reduction was compensated for by matrix polysaccharides rather than by cellulose (left column). The distinction between the different mutants and the wild-type is visualized by different colors. Scatterplots (middle and right columns) illustrate the relations between saccharification without and with acid pretreatment, respectively, and the different cell wall polymers (lignin, cellulose, and matrix polysaccharides). The Pearson correlation coefficient and its corresponding p-value are given in the upper left corner of each scatterplot. [file 1754-6834-6-46-S2.pdf]
